# Rituximab-Induced Colitis and Esophagitis in a Patient With Granulomatosis With Polyangiitis

**DOI:** 10.7759/cureus.38207

**Published:** 2023-04-27

**Authors:** William K Boateng, Fomengia Joseph Nkeangu, Manlio H Castillo, Valentin Marian, Tingliang Shen

**Affiliations:** 1 Internal Medicine, Jersey City Medical Center, Jersey City, USA; 2 Rheumatology, Jersey City Medical Center, Jersey City, USA; 3 Pathology, Jersey City Medical Center, Jersey City, USA

**Keywords:** immune-mediated colitis, side effects of medical treatment, rituximab, colitis, granulomatosis with polyangiitis (gpa)

## Abstract

Granulomatosis with polyangiitis (GPA) is a small vessel vasculitis that affects many organ systems with varying disease severity. GPA commonly affects the sinuses and lung parenchyma. However, GPA can affect the gastrointestinal tract and may present as colitis. Immunosuppressive therapy, like rituximab (RTX), is used for the management of this disease. Rituximab is generally well-tolerated but has rare side effects that have been shown to mimic colitis in inflammatory diseases. Our case is a 44-year-old female with a history of GPA who presented with dysphagia, abdominal pain, and diarrhea. The patient received a maintenance dose of RTX six months before the presentation. The patient was seronegative for anti-neutrophilic cytoplasmic antibodies against proteinase 3 (PR3 ANCA). Infectious etiology was ruled out. Esophagogastroduodenoscopy (EGD) and colonoscopy showed esophageal bleeding ulcers and diffuse colonic inflammation, respectively. Pathology was consistent with esophagitis and colitis. Colonic mucosal biopsy failed to show evidence of vasculitis. The patient was treated with sucralfate and intravenous pantoprazole with an improvement in the symptoms. The repeat endoscopy on an outpatient basis showed the patient had full mucosal healing, including histological healing. Our patient likely had rituximab-induced colitis and esophagitis.

## Introduction

Granulomatosis with polyangiitis (GPA) is a rare blood vessel disease affecting many organ systems with varying disease severity. Immunosuppressive therapy has been the cornerstone in the management of this disease, with a significant decrease in mortality following appropriate treatment. However, these agents are not without side effects, thereby posing a diagnostic dilemma between medication side effects and disease progression. Rituximab (RTX) is known to be well-tolerated, but on rare occasions, RTX-induced colitis can present as a complication of treatment [1]. Esophagitis caused by RTX is also an uncommon complication [2]. There are unique occasions where GPA presents gastrointestinal manifestations such as esophagitis and colitis [3,4]. When a patient with GPA who was treated with RTX develops these symptoms, it is often difficult to discern the underlying etiology.

Herein, we describe a case where RTX induces colitis and esophagitis in a patient with a history of GPA.

## Case presentation

We present a 44-year-old female with a history of granulomatosis with polyangiitis (GPA) that came in after a five-week history of abdominal pain and diarrhea.

The patient was diagnosed with limited GPA in 2017. She had a GPA history that was significant for lung granulomatous inflammation and positive anti-neutrophilic cytoplasmic antibodies against proteinase 3 (PR3 ANCA). She received treatment with an anti-CD20 antibody, rituximab (RTX). Her symptoms improved and eventually achieved remission.

On presentation, the patient stated that her abdominal pain was postprandial, crampy, located in the periumbilical region, and usually preceded diarrhea. She reported having between three and five episodes of mucoid non-bloody stools, anorexia, and a nine-pound weight loss in two weeks. Her symptoms were associated with a sensation of food getting stuck in her throat and pain when she swallowed two days before admission. She denied fever, nausea, vomiting, or dark-colored stool. She reported worsening sinus congestion for which she had been seeing an otolaryngologist. She was referred to a gastroenterologist because of the dysphagia one week before the presentation. The patient states that an esophagogram was done and was shown to be within normal limits.

**Figure 1:**
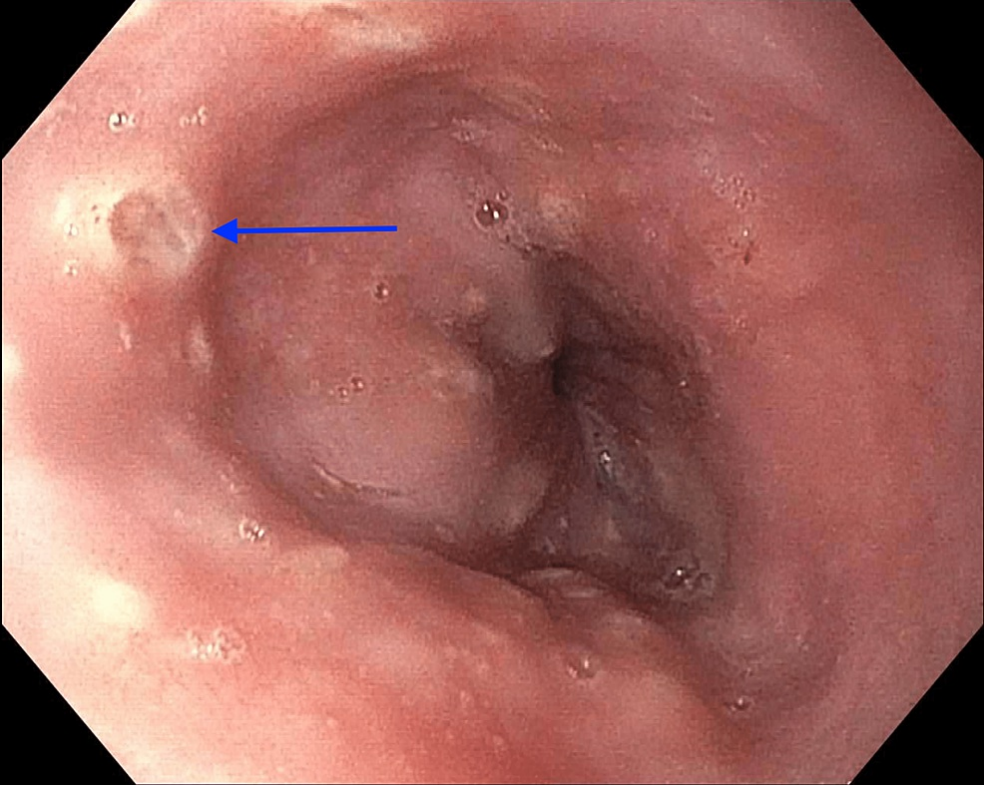
EGD finding of large bleeding ulcers in the third part of the esophagus EGD: esophagogastroduodenoscopy

**Figure 2:**
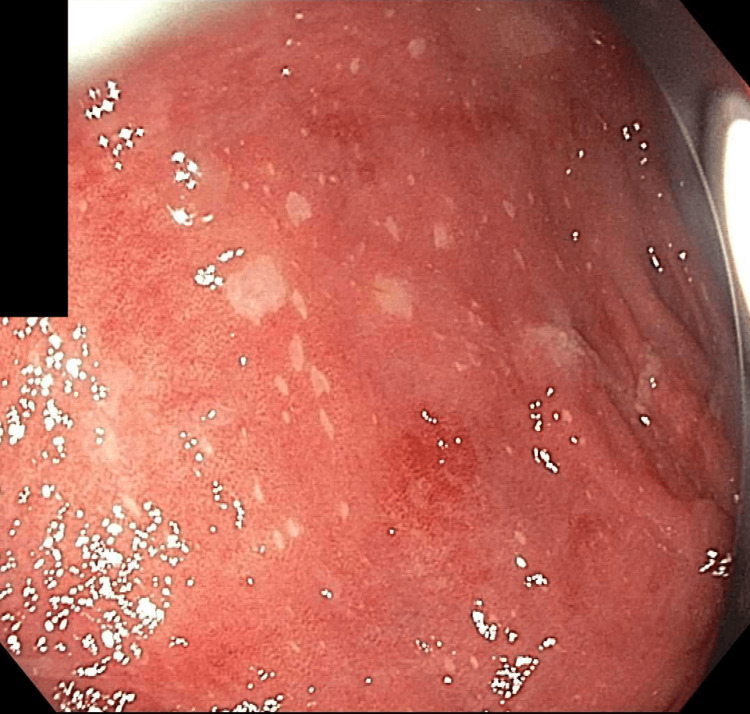
Colonoscopy finding of diffuse mild inflammation in the sigmoid colon

At this time, the patient’s gastrointestinal symptoms persisted, and she decided to come to the emergency department (ED) for evaluation. In the ED, a computerized tomography scan showed colonic wall thickening, suggestive of infectious or inflammatory colitis. It also showed a patulous esophagus and possible mild reflux esophagitis. After admission, the patient was treated with oral sucralfate and intravenous pantoprazole for esophagitis. Gastroenterology and rheumatology were consulted. Laboratory investigations showed a negative anti-neutrophilic cytoplasmic antibody (ANCA) profile, blood and stool culture, herpes simplex virus (HSV), and human immunodeficiency virus (HIV) serology. C-reactive protein, erythrocyte sedimentation rate, and stool calprotectin were all elevated. Esophagogastroduodenoscopy (EGD) showed non-bleeding esophageal ulcers as depicted in Figure 1. A colonoscopy showed pancolitis with diffuse mild inflammation found in the entire colon (Figure 2).

**Figure 3:**
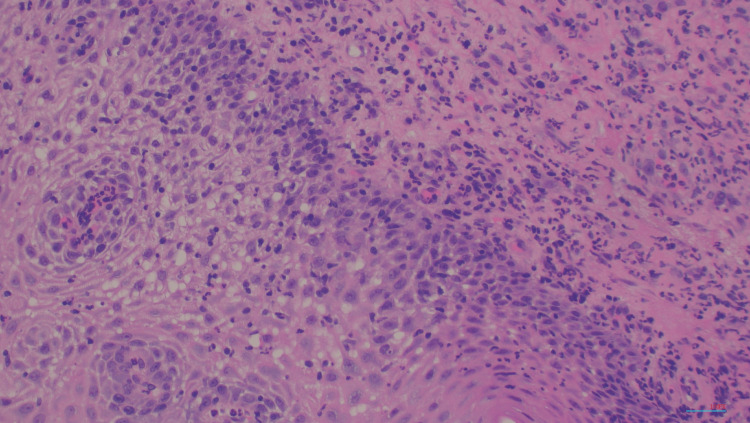
H&E histology stain, 100x magnification Esophageal squamous mucosa with acute and chronic esophagitis showing neutrophils and infiltrating squamous epithelium and underlying stroma H&E: hematoxylin and eosin

**Figure 4:**
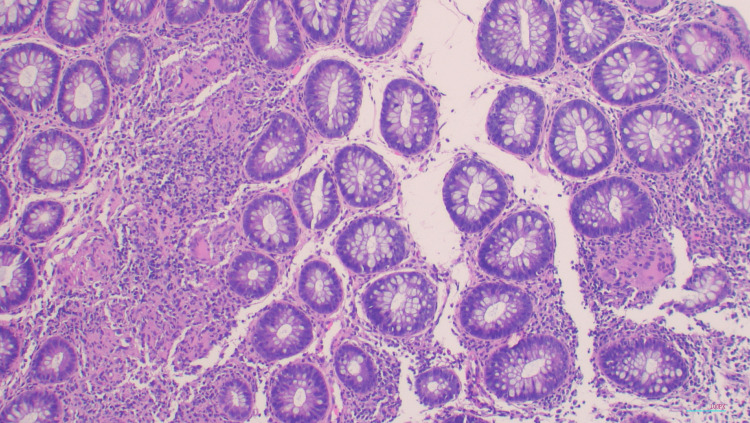
H&E histology stain, 100x magnification Transverse mucosa shows many non-caseating granulomas forming coalescent lesions in lamina propria and one single granuloma in the right mid-field. H&E: hematoxylin and eosin

**Figure 5:**
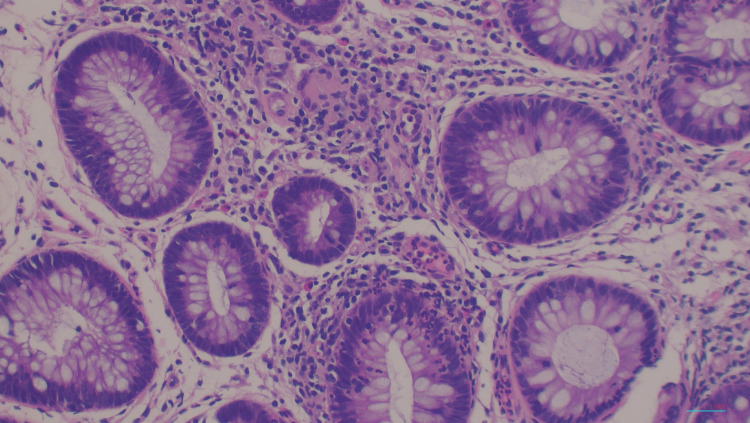
H&E histology stain, 100x magnification Rectal mucosa with active colitis showing cryptitis and adjacent non-caseating granulomas H&E: hematoxylin and eosin

A biopsy of the esophagus showed acute and chronic esophagitis. It was negative for Cytomegalovirus and HSV I/II and negative for fungi by periodic acid-Schiff stain as depicted in Figure 3. A biopsy of the colon showed active colitis and granulomas depicted in Figure 4. There was also encompassing cryptitis throughout most of the colon. No necrosis or vasculitis was seen as depicted in Figure 5.

The patient had a favorable response to symptomatic treatment with intravenous pantoprazole and sucralfate and was discharged home with significant improvement. In subsequent outpatient visits, she remained asymptomatic. The repeat endoscopy was benign and showed she had full mucosal healing, including histological healing.

## Discussion

GPA is a small vessel vasculitis that affects multiple organs. The full scope of the pathogenesis of GPA is still unknown. Genetic, environmental, and epigenetic factors all play a role. Anti-neutrophil cytoplasmic antibodies (ANCA) that primarily target proteinase 3 (PR3), which are found within neutrophils, are believed to play a role in the pathophysiology. The evidence is circumstantial, and opinions are divided among experts.

GPA is commonly treated with RTX, which is a monoclonal antibody that targets the CD20 marker present in most of the mature B lymphocytes. RTX was approved by the United States Food and Drug Administration (FDA) for the treatment of ANCA-related vasculitis after a successful randomized control trial [5]. It is generally well-tolerated, but on rare occasions, treatment results in adverse reactions. De novo colitis was associated with RTX infusion in a study conducted by Eckmann et al. [1]. A few patients with diseases treated with RTX developed colitis resembling inflammatory bowel disease. Our patient’s last exposure to RTX was six months before the presentation, and she received maintenance-level dosing (a single infusion of 500 mg). We hypothesize that at the time of presentation in the ED, the immunologic activity of RTX would have been at the tail end of any significant immunomodulatory effects. However, according to a study done by Mallepally et al., RTX-induced colitis should be considered in patients who received RTX treatment with GI symptoms for up to two years after patients receive the last dose of rituximab [6]. There are reported cases of colitis presentation from immediately after infusion to two years after RTX administration [3,7-12]. It is important to note that most case reports of colitis induced by RTX were either diagnosed as Crohn’s disease or ulcerative colitis based on clinical presentation and histology [7-12]. Our patient’s histology showed cryptitis and non-caseating granulomas, similar to the pathology found in Crohn’s disease. The pathogenesis of RTX-induced colitis may involve the role RTX plays in the depletion of CD20+ B cells. B and T lymphocytes are responsible for the mucosal immunoregulation in the GIT, increasing immune tolerance [11]. The colitis found after RTX may be due to CD20+ B cell depletion and infiltration of T lymphocytes in the mucosa, leading to immune dysregulation [6,11]. We found only one reported case of RTX causing esophagitis [2]. The patient in the case had an esophageal ulcer that was ultimately responsive to steroids after a failed trial of proton pump inhibitors.

During our patient’s course, we were able to rule out infectious causes of colitis and esophagitis. Our patient had worsening sinusitis for two days before the presentation. There may have been a possibility that colitis, esophageal ulcers, and worsening sinusitis emerged as a GPA flare after the therapeutic effects of RTX faded. Serology tests, however, were negative for PR3 and anti-myeloperoxidase ANCA. Chest imaging did not reveal any pulmonary parenchymal disease that was indicative of this patient’s usual flare. The patient’s nodules and lung interstitials were improved. The colonic mucosal biopsy failed to show evidence of vasculitis or necrosis.

The clinical manifestations of GPA vary greatly. When our patient was initially diagnosed, the GPA symptoms presented as sinusitis, pulmonary nodules, and infiltrates. These symptoms are among the most common presentations in GPA [13]. Gastrointestinal tract (GIT) involvement in GPA is uncommon and can be an elusive presentation in the setting of RTX treatment.

GPA can affect any part of the gastrointestinal tract. Though uncommon, there are reports of patients with GPA presenting with colitis and esophagitis [2,14-17]. Some clinical manifestations include bloody diarrhea, melena, and abdominal pain [15,17]. Endoscopy often reveals the inciting damage along with a biopsy to determine the pathological characteristics of the inflammation. There are only a few reported cases of esophageal involvement with GPA. Similarly, in these cases, patients presented dysphagia, odynophagia, and reflux symptoms [4,16]. Moreover, patients with GPA tend to have evidence of active vasculitis elsewhere and are not just limited to the GIT. In all GPA cases that we reviewed, PR3 ANCA titers were positive [4,14-17].

## Conclusions

The etiology of colitis and esophageal ulcers, in this case, is unique. Gastrointestinal manifestations in GPA are rare and all other etiologies, such as treatments and infections, must be ruled out. Exposure to RTX has been shown to cause inflammation in the gastrointestinal tract at variable times of onset. It is important to be aware of the potentially rare side effects of immunosuppressants, as some can mimic the very disease they are intended to treat.

## References

[1] Eckmann JD, Chedid V, Quinn KP, Bonthu N, Nehra V, Raffals LE (2020). De novo colitis associated with rituximab in 21 patients at a tertiary center. Clin Gastroenterol Hepatol.

[2] Balagoni H, Smith AK, Chaudhari D, Young MF (2007). A tough pill to swallow: a case of rituximab-associated esophageal ulcers. Am J Gastroenterol.

[3] Barreiro Alonso E, Álvarez Álvarez A, Tojo González R, de la Coba Ortiz C (2019). Rituximab-associated colitis. Gastroenterol Hepatol.

[4] Fallows GA, Hamilton SF, Taylor DS, Reddy SB (2000). Esophageal involvement in Wegener's granulomatosis: a case report and review of the literature. Can J Gastroenterol.

[5] Stone JH, Merkel PA, Spiera R (2010). Rituximab versus cyclophosphamide for ANCA-associated vasculitis. N Engl J Med.

[6] Mallepally N, Abu-Sbeih H, Ahmed O, Chen E, Shafi MA, Neelapu SS, Wang Y (2019). Clinical features of rituximab-associated gastrointestinal toxicities. Am J Clin Oncol.

[7] Shahmohammadi S, Sahraian MA, Shahmohammadi A, Doosti R, Zare-Mirzaie A, Naser Moghadasi A (2018). A presentation of ulcerative colitis after rituximab therapy in a patient with multiple sclerosis and literature review. Mult Scler Relat Disord.

[8] Morita K, Shibano T, Maekawa K, Hattori M, Hida N, Nakamura S, Takeshima Y (2019). Crohn's disease following rituximab treatment in a patient with refractory nephrotic syndrome. CEN Case Rep.

[9] Cavalcanti E, Armentano R, Lolli I (2020). Crohn's disease following rituximab treatment for follicular lymphoma in a patient with synchronous gastric signet ring cells carcinoma: a case report and literature review. Cancer Res Treat.

[10] Fraser D, Boyle S, Amft N (2016). Perianal Crohn disease after treatment with rituximab for active granulomatosis with polyangiitis. J Rheumatol.

[11] Varma P, Falconer J, Aga A, Prince HM, Pianko S (2017). Rituximab-induced Crohn's disease. Scand J Gastroenterol.

[12] Ardelean DS, Gonska T, Wires S (2010). Severe ulcerative colitis after rituximab therapy. Pediatrics.

[13] Hoffman GS, Kerr GS, Leavitt RY (1992). Wegener granulomatosis: an analysis of 158 patients. Ann Intern Med.

[14] Pan SW, Wang C, Zhang X (2018). A rare endoscopic appearance of granulomatosis with polyangiitis involving the intestine: a case report. BMC Gastroenterol.

[15] Sinnott JD, Matthews P, Fletcher S (2013). Colitis: an unusual presentation of Wegener's granulomatosis. BMJ Case Rep.

[16] Arista S, Sailler L, Astudillo L (2005). Relapsing esophageal and gastric ulcers revealing Wegener's granulomatosis. Am J Med.

[17] Amber KT, Bunyan A, Lee JJ, Duvvuri A (2016). Granulomatosis with polyangiitis (GPA) initially presenting as sigmoiditis with the later development of spontaneous subcapsular hematoma. Int J Colorectal Dis.

